# Impact of flour fortification with calcium on calcium intake: a simulation study in seven countries

**DOI:** 10.1111/nyas.14550

**Published:** 2021-01-11

**Authors:** Gabriela Cormick, Ana Pilar Betran, Iris B. Romero, María Nieves García‐Casal, Surya M. Perez, Luz Gibbons, José M. Belizán

**Affiliations:** ^1^ Department of Mother and Child Health Research Institute for Clinical Effectiveness and Health Policy (IECS‐CONICET) Buenos Aires Argentina; ^2^ Departamento de Salud Universidad Nacional de La Matanza (UNLAM) San Justo Argentina; ^3^ UNDP/UNFPA/UNICEF/WHO/World Bank Special Programme of Research, Development and Research Training in Human Reproduction (HRP), Department of Sexual and Reproductive Health and Research World Health Organization Geneva Switzerland; ^4^ Department of Nutrition and Food Safety World Health Organization Geneva Switzerland

**Keywords:** calcium intake, fortification, flour, simulation, adequacy, upper limit, calcium recommendations

## Abstract

Calcium intake is low in many countries, especially in low‐income countries. Our objective was to perform a simulation exercise on the impact, effectiveness, and safety of a flour fortification strategy using the Intake Modelling, Assessment, and Planning Program. Modeling of calcium fortification scenarios was performed with available dietary intake databases from Argentina, Bangladesh, Italy, the Lao People's Democratic Republic (Lao PDR), Uganda, Zambia, and the United States. This theoretical exercise showed that simulating a fortification with 156 mg of calcium per 100 g of flour would decrease the prevalence of low calcium intake, and less than 2% of the individuals would exceed the recommended calcium upper limit (UL) in Argentina, Italy, Uganda, and Zambia. Bangladesh and the Lao PDR showed little impact, as flour intake is uncommon. By contrast, in the United States, this strategy would lead to some population groups exceeding the UL. This exercise should be replicated and adapted to each country, taking into account the updated prevalence of calcium inadequacy, flour consumption, and technical compatibility between calcium and the flour‐type candidate for fortification. A fortification plan should consider the impact on all age groups to avoid the risk of exceeding the upper levels of calcium intake.

## Graphical abstract

This study shows that in some low‐ and middle‐income countries (LMICs), flour fortification would, in theory, increase calcium intake without posing the risk of excess of calcium intake in the population. However, in two LMICs, flour intake was so low that the impact on calcium intake was negligible, and fortification of other widely consumed foods should be considered. Results shown in this study are limited to the countries selected, but it can be hypothesized that similar results would be obtained in other LMICs. The analysis performed in this study is required in any country with the most updated information before implementing a fortification strategy.

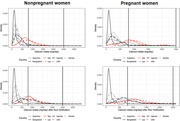

## Introduction

Adequate calcium intake is associated with bone health, and increasing evidence shows the link between adequate calcium intake and lower blood pressure, particularly among young people, the prevention of hypertensive disorders of pregnancy, and lower blood pressure in the progeny of mothers taking sufficient calcium during pregnancy.^1^, ^2^, ^3^, ^4^, ^5^ An adequate calcium intake has also been shown to lower cholesterol values and prevent renal stones and colorectal adenomas.^6^, ^7^, ^8^, ^9^, ^10^

Calcium intake is low in many countries in the world, especially in low‐ and middle‐income countries (LMICs), though within certain high‐income countries (HICs), low calcium intake can also be found within some population age groups.^5^, ^11^, ^12^, ^13^ To achieve recommended intake, diets with calcium‐rich foods are ideal; however, a change in dietary habits is difficult to achieve in a short period of time.^14^ Another strategy to increase calcium intake is through calcium supplements, but adherence to supplements is usually low.^5^

Food fortification strategies for calcium may be an effective and cost‐effective alternative strategy.^15^, ^16^, ^17^ Preliminary steps to assess food fortification as a strategy include to determine the appropriate food (vehicle) and calcium salt (fortificant) as well as the fortification level necessary to improve calcium intake without putting any population group at risk of excessive intake.^18^

In order to define the fortification level, representative data on food and nutrient intake for the aimed population are required so as to identify the level of nutrient inadequacy in the population and the appropriate food vehicle that is sufficiently consumed to cover this gap.^18^ The feasibility of the industrial process and the compatibility of calcium, which is very bulky, with the fortification vehicle is crucial to warrant a final product correctly fortified and well accepted by the population. Simulation methods may be used to estimate the impact of different fortification levels on the distribution of nutrient intake of each population group that will be reached by the fortification strategy. Simulation allows estimating the reduction of nutrient inadequacy for each population group as well as the percentage of individuals with excess intake.^19^, ^20^, ^21^

Fortification of wheat and maize flour has been used for many years to increase the intake of iron, folic acid, vitamin B_12_, vitamin A (VA), and zinc, although the evidence of its effect on clinical outcomes is not always available.^22^, ^23^, ^24^ The United Kingdom has mandatory fortification of flour with calcium since 1943 (between 94 and 156 mg of calcium per 100 g of white wheat flour) with the aim to improve calcium intake in the war times when dairy products were scarce. This policy was reinforced in 1998 after a review had found that stopping this mandatory fortification of flour with calcium would increase the proportion of the population with inadequate calcium intake.^25^ The review showed that stopping calcium addition to flour would have no significant effect in the calcium intake of children younger than 10 years; however, it would increase the percentage of older children with inadequate calcium intake from 15% to 21% in girls aged 11–18 years, and from 8% to 12% in boys aged 11–18 years. In adults, the percentages of inadequacy would increase from 3% to 4% for men and from 6% to 9% for women; and from 1% to 3% for men and from 3% to 5% for women in older people if added calcium was removed.^25^

The objective of this manuscript is to present a theoretical simulation exercise of the impact of flour fortification with calcium on calcium intake adequacy in seven countries: Argentina, Bangladesh, Italy, the Lao People's Democratic Republic (LAO PDR), Uganda, the United States, and Zambia.^11^ These countries show a diversity of income and development, including LMICs from South America, Africa, and Asia, and two HICs.

## Materials and methods

We searched for available national or subnational dietary assessment databases collected through 24‐h recalls or dietary records with individual information of food amount intake with free public access. In our search, we found dietary intake databases of 24‐h recalls from Argentina, Bangladesh, the Lao PDR, Uganda, the United States, and Zambia, and of self‐recorded food records from Italy, and thus, we included these countries in our analysis. Argentina, Bangladesh, the Lao PDR, Uganda, and Zambia are countries in regions with very low calcium intake (below 400 mg/day), whereas the United States and Italy are countries with adequate calcium intake.^11^ Databases from Bangladesh, Italy, the Lao PDR, Uganda, and Zambia were obtained from the FAO/WHO Global Individual Food consumption data Tool (FAO/WHO GIFT), an open‐access online platform hosted by the FAO and supported by the WHO.^26^ The database of Argentina was obtained from the Ministry of Health, and the U.S. database was obtained from the Centers for Diseases Control and Prevention (CDC) website.^27^, ^28^

We used the Intake Modelling Assessment Program (IMAPP) developed by the Iowa University, a computer program that allows running different simulation scenarios of nutrient intake using the information of daily intake of population groups and estimates the best amount of a fortificant to be added to a food vehicle in order to decrease the level of nutrient inadequacy without exceeding the recommended upper limits (ULs).^19^, ^20^ The program requires the use of individual dietary information; therefore, we searched for 24‐h recalls or dietary records. We simulated the shifts in the distribution of calcium intake necessary so that the majority of population groups improved their calcium intake and achieved a mean intake closer to their requirement without exceeding the recommended UL. For each database, we evaluated the effectiveness and safety of flour fortification with 156 mg of calcium per 100 g of flour.^19^ On the basis of the extensive experience of mandatory flour fortification in the United Kingdom, we selected 156 mg of calcium per 100 g of flour as this is the maximum fortification level allowed in the United Kingdom, and thus, a feasible level to fortify.^29^ Effectiveness was measured as the percentage of individuals below the estimated average intake (EAR) and safety as the percentage of individuals exceeding the UL of their corresponding age‐specific population subgroup. We used the default harmonized dietary reference values in the IMAPP program that are mainly a compilation of EARs from IOM DRIs, RNIs from the FAO/WHO tables. For a proper simulation, it would be ideal to use each country's dietary references; however, not all selected countries have their own references, and besides, it would be difficult to compare results in this exercise.

The EAR was defined as a nutrient intake value that is estimated to meet the requirement of half of the healthy individuals in a group.^18^ The ULs reflect the maximum daily intake levels at which no risk of adverse health effects is expected for almost all individuals in the general population, including sensitive individuals, when the nutrient is consumed over long periods of time. In other words, the UL is the highest usual intake level of a nutrient that poses no risk of adverse effects.^30^

### Population

#### Argentina

Data were obtained from the first Health and Nutrition National Survey (abbreviated as ENNyS in Spanish) carried out by the Ministry of Health in Argentina between 2004 and 2005.^27^ Participants were selected using a probabilistic complex sample design, including different socioeconomic levels from both large and small cities of all provinces of Argentina representing the whole population.^31^ Data were collected using a single 24‐h recall. The survey included weights that were used in the IMAPP analysis.

The survey included 1338 children aged 0 to less than 9 years, 6605 women aged 19 to less than 50 years, and 1610 pregnant women (PW) 14 to less than 45 years.

#### Bangladesh

Consumption data from the initial survey of a HarvestPlus multistage research program to determine the potential impact of zinc‐biofortified rice on the zinc and health status among children in Bangladesh were used to obtain consumption data. The original study was conducted in collaboration with the University of California, Davis, and the International Centre for Diarrheal Disease Research, Bangladesh (ICDDR). Data were collected in two rural rice‐producing regions: in Trishal from late October 2007 through early May 2008, and in Pirgaccha from late January through June 2008. Dietary information was collected primarily by direct observation and weighing of food preparation and consumption.^26^

According to the IOM age categories, there were 224 nonpregnant women (NPW) aged 18–70 years and 236 PW aged 19–51 years. The survey also included girls aged 14–19 years and women aged 51 to less than 71 years; however, the analysis of these age groups was not possible owing to the low number of cases.^32^

#### Italy

Data were obtained from the third national survey L'indagine Nazionale sui Consumi Alimentari in Italia (abbreviated as INRAN‐SCAI in Italian) performed by the Italian Consiglio per la Ricerca in Agricoltura E L'analisi Dell'economia Agrarian (abbreviated as CREA in Italian) from October 2005 to December 2006.^33^ The survey covered all seasons and included a sample representative of the Northwest, Northeast, Center, South, and the Islands of Italy. Consumption of all foods, beverages, food supplements, and medicines was self‐recorded by subjects for 3 consecutive days on hard‐copy diaries structured by meal. For our analysis, we used the first and third survey days.

The survey included 3323 individuals: 1501 males and 1793 females; however, some did not have all required data, and finally, 3269 were analyzed, including 377 children aged 0 to less than 14 years, 28 PW aged 19 to less than 51 years, 1584 NPW aged 19 to less than 97 years, and 1280 men aged 19 to less than 92 years.

#### The Lao PDR

Data were obtained from the National Food Consumption Survey Lao PDR performed in rural and urban settings from December 2016 to May 2017.^26^ The survey included 2045 individuals: 870 males and 1175 women. Owing to the low sample size, NPW aged 71 or above and boys aged 9 to less than 19 years were excluded from this analysis.^32^ A total of 1919 individuals were analyzed in this study: 1048 children aged 3 months to less than 14 years, 319 NPW older than 14 years, 285 PW older than 16 years, and 267 men older than 19 years.

#### Uganda

Dietary intakes from Uganda were obtained from the baseline survey of the HarvestPlus Reaching End Users (REU) Orange‐Fleshed Sweet Potato (OFSP) project performed in three rural regions of Eastern and Central Uganda: Bukedea, Kamuli, and Mukono. This cross‐sectional study took place from January 1 to December 31, 2007. The project aimed at inducing broad OFSP adoption to increase VA intakes among women in Uganda.^26^ The survey included 577 women of reproductive age. After classifying participants into the IOM age categories for recommended dietary values, we found that only three girls were younger than 19 years, so they were excluded from the analysis. Nine of those had missing data; they were also excluded. A total of 565 women were included in the dietary intake analysis: 270 NPW aged 20–67 years and 295 PW aged 19–48 years.

#### The United States

The data source for the United States was 2016 nationally representative, cross‐sectional survey of the noninstitutionalized U.S. population (NHANES) administered by the National Center for Health Statistics within the CDC.^34^ The survey has a stratified, multistage probability cluster sampling design. The survey included weights that were used in the IMAPP analysis.

The survey included 9971 individuals, although only 8339 had data on calcium intake: 2443 children aged 0 to less than 19 years, 2974 women aged 19 to less than 80 years, 2859 men aged 19 to less than 80 years, and 63 PW 20 to less than 42 years.

#### Zambia

Dietary intakes from Zambia were obtained from the initial survey of the HarvestPlus nutritional survey. This survey was carried out in Nyimba District in Eastern Province and Mkushi District in Central Province with the collaboration with the National Food and Nutrition Commission (Lusaka, Zambia) and the Tropical Diseases Research Centre (Ndola, Zambia) from April 30, 2007 to December 29, 2009. The main goal of the survey was to obtain adequate background nutritional information among preschool children from rural Zambia to assess the potential impact of food‐based interventions to improve VA status, including proVA‐biofortified maize.^26^

The survey included 454 children aged 1 to less than 9 years, 145 NPW aged 19 to less than 50 years, and 186 PW aged 17 to less than 51 years. Other groups (infants and females aged 14 to less than 19 years and/or 51 to less than 71 years, pregnant or not) were not included in the analysis because of an insufficient number.^32^

### Analysis

Usual calcium intake was estimated using the daily intake information from individuals in each of the selected databases and available age groups. We followed the methodology to assess the usual calcium intake to obtain adjusted intake distributions.^35^ For those populations that had repeated information in a nonconsecutive day, we used these repeated recalls to calculate the day‐to‐day variability of calcium intake using the IMAPP. The variability was then used to adjust the calcium intake distribution of one single day in order to obtain an estimated distribution of the usual calcium intake. For populations with no repeated information, we estimated the usual calcium intake using a default external variance ratio for calcium provided by the IMAPP.

The IMAPP was used to assign each population group the corresponding calcium EAR and UL needed to calculate the baseline prevalence of inadequate calcium intake as the proportion of individuals in the group with usual calcium intake below the age‐specific EAR and the proportion of individuals with usual calcium intakes above the age‐specific UL, using values defined by the IMAPP.^36^ We then calculated the initial gap defined as the estimated amount of calcium that should be added to flour in order to achieve the “target prevalence of inadequate intakes.” The initial gap was calculated as the difference in mg per day between the EAR for the target population group and the usual calcium intake corresponding to the desired prevalence for that group. We used a target prevalence of 50% when the prevalence of inadequate intake was above 50%, 10% when it was between 10% and 50%, and 0% when it was less than 10%.

Subsequently, for each country database, the flour content of each of the foods that contained wheat, rice, maize, or any kind of flour was estimated. For example, if the food item in the database was flour, the flour content was 100%. However, if the food item was bread, we calculated the percentage of flour content in that bread. We used published documents to estimate these percentages of flour, for example, the UK Scientific Advisory Committee document on nutrition and nutritional implications of repealing the UK bread and flour regulations.^25^ However, when the food item was not in these documents, we estimated the content of flour from local recipes, corresponding food websites, or food labels.

For each country, a distribution of the consumption of all flours was made. When the food item contained a mix of flours, we calculated the amount of flour in that food as the sum of all flours present in the food. For example, if the food had 30% of rice flour and 40% of wheat flour, we calculated that all flour will be fortified, and the amount of flour was 70%. In real‐case scenarios, it would be necessary to assess the feasibility of the fortification of each flour source.

For the analysis of flour content, all supplements or medicines in the form of tablets or pills that may contain starch were not contemplated, whereas dietary supplements in the form of powder, shakes, or liquid ready to drink were included. Finally, using the IMAPP, the adjusted calcium intake distributions for each age group after simulating the addition of 156 mg of calcium to 100 g of flour were estimated to assess the impact on inadequate calcium intake and risk of excess.

## Results

For each country included in this analysis, Tables 1, 2, 3, 4, 5, 6, 7 show the number of individuals for each age group, the percentage of individuals who reported eating any food containing flour (we used the first dietary assessment if they had more than one), and the average flour intake. The percentage of the population consuming flour varied among countries going from a very low percentage in Bangladesh and the Lao PDR to most of the population consuming flour like in Zambia, Italy, the United States, and Argentina.

**Table 1:** Results of the simulation for Argentina: hypothetical changes in usual (adjusted) calcium intake after addition of 156 mg of calcium per 100 g of flour

				Flour consumption			Calcium intake				Calcium intake after fortification with 156 mg in 100 g flour			
Group (age in years)	N	EAR	UL	% population consuming flour	Mean flour intake (mg)	SD	Mean (mg)	SD	<EAR (%)	>UL (%)	Mean (mg)	SD	<EAR (%)	>UL (%)
**Children**														
0.5 ≤ age < 1	2110	270	1500	80.9	22.2	25.4	609.1	460.4	17.5	1.5	644.0	353.7	14.4	1.8
1 ≤ age < 4	7787	400	2500	96.5	53.6	42.1	752.2	323.4	13.4	0.0	835.7	329.8	8.5	0.0
4 to < 9	3480	640	2500	98.4	89.1	61.1	683.3	260.6	46.2	0.0	821.3	266.8	26.0	0.0
**Nonpregnant women**														
9 ≤ age < 14	864	1100	3000	99.2	120.5	72.8	460.7	167.6	99.9	0.0	649.6	188.1	98.3	0.0
14 ≤ age < 19	1122	1100	3000	98.3	116.1	81.1	438.8	198.2	99.4	0.0	619.8	230.6	96.5	0.0
19 ≤ age < 31	2112	800	2500	97.8	103.6	75.3	402.6	168.1	97.6	0.0	564.7	193.1	88.4	0.0
31 ≤ age < 51	2507	800	2500	82.4	87.9	64.5	361.6	180.2	97.6	0.0	499.4	200.1	92.0	0.0
**Pregnant women**														
14 ≤ age < 19	197	1100	3000	98.0	137.2	100.9	470.6	246.7	97.8	0.0	684.5	287.0	91.4	0.0
19 ≤ age < 31	963	800	2500	98.8	123.0	82.2	490.6	240.1	89.0	0.0	682.8	274.1	70.8	0.0
31 ≤ age < 51	450	800	2500	98.7	107.8	82.4	491.3	244.7	88.4	0.0	659.8	275.1	73.0	0.0

note: Shown for each age group are the number of individuals included in the analysis, dietary reference values for calcium (EAR and UL), percentage of individuals who consumed flour, mean flour intake taking into account all the individuals in the group and SD, calcium intake, adequacy of calcium intake, and calcium intake after fortifying flour with 156 mg of calcium per 100 g of flour.

EAR, estimated average requirement; UL, the upper limit for calcium according to the dietary reference intakes of the Institute of Medicine Food and Nutrition Board.

**Table 2:** Results of the simulation for Bangladesh: hypothetical changes in usual (adjusted) calcium intake after addition of 156 mg of calcium per 100 g of flour

					Flour consumption			Calcium intake				Calcium intake after fortification with 156 mg in 100 g flour			
Group (age in years)	N round 1	N round 2	EAR	UL	% population consuming flour	Mean flour intake (mg)	SD	Mean (mg)	SD	<EAR (%)	>UL (%)	Mean (mg)	SD	<EAR (%)	>UL (%)
**Nonpregnant women**															
19 ≤ age < 31	157	157	800	2500	6.4	1.9	6.8	160.8	55.6	100.0	0.0	164.1	58.4	100.0	0.0
31 ≤ age < 51	67	67	800	2500	13.4	3.1	13.2	151.8	53.7	100.0	0.0	156.8	58.7	100.0	0.0
**Pregnant women**															
19 ≤ age < 31	174	173	800	2500	8.6	1.6	6.5	150.2	58.8	100.0	0.0	152.1	61.1	100.0	0.0
31 ≤ age < 51	62	62	800	2500	9.7	1.5	5.9	143.2	80.3	100.0	0.0	146.0	88.4	99.9	0.0

note: Shown for each age group are the number of individuals included in the analysis, dietary reference values for calcium (EAR and UL), percentage of individuals who consumed flour, mean flour intake taking into account all the individuals in the group and SD, calcium intake, adequacy of calcium intake, and calcium intake after fortifying flour with 156 mg of calcium per 100 g of flour.

EAR, estimated average requirement; UL, the upper limit for calcium according to the dietary reference intakes of the Institute of Medicine Food and Nutrition Board.

**Table 3:** Results of the simulation for Italy: hypothetical changes in usual (adjusted) calcium intake after addition of 156 mg of calcium per 100 g of flour

					Flour consumption			Calcium intake				Calcium intake after fortification with 156 mg in 100 g flour			
Group (age in years)	N round 1	N round 2	EAR	UL	% population consuming flour	Mean flour intake (mg)	SD	Mean (mg)	SD	<EAR (%)	>UL (%)	Mean (mg)	SD	<EAR (%)	>UL (%)
**Children**															
0.5 to < 1	9	9	270	1500	100.0	28.5	20.2	600.0	147.3	1.7	0.0	639.1	145.5	1.0	0.0
1 ≤ age < 4	53	53	400	2500	100.0	64.0	37.1	728.6	180.5	1.9	0.0	822.0	172.9	0.3	0.0
4 ≤ age < 9	145	145	640	2500	99.3	109.0	50.4	725.9	210.6	37.2	0.0	890.9	229.1	12.6	0.0
**Pregnant women**															
19 ≤ age < 31	4	4	800	2500	100.0	105.5	48.2	1009.1	181.2	13.1	0.0	1299.7	170.6	0.6	0.0
31 ≤ age < 51	24	24	800	2500	100.0	146.3	78.2	822.0	267.6	51.0	0.0	1029.1	297.1	22.9	0.0
**Nonpregnant women**															
9 ≤ age < 14	83	83	1100	3000	100.0	125.7	52.9	791.2	247.8	88.9	0.0	992.4	267.6	69.1	0.0
14 ≤ age < 19	84	84	1100	3000	100.0	139.1	64.5	819.5	187.0	92.3	0.0	1032.5	216.5	64.8	0.0
19 ≤ age < 31	261	261	800	2500	99.6	118.0	58.9	716.4	237.5	68.4	0.0	905.2	264.4	37.3	0.0
31 ≤ age < 51	551	551	800	2500	99.3	114.1	57.5	734.9	236.3	65.7	0.0	914.6	270.3	36.6	0.0
51 ≤ age < 71	482	482	1000	2000	98.8	104.0	50.8	756.0	243.9	84.7	0.0	914.8	260.9	66.6	0.1
age ≥ 71	206	206	1000	2000	100.0	106.3	53.9	799.7	228.4	81.8	0.0	963.8	244.5	59.0	0.0
**Male**															
9 ≤ age < 14	83	83	1100	3000	98.5	151.4	62.6	881.9	325.0	78.0	0.0	1106.2	354.6	53.9	0.0
14 ≤ age < 19	68	68	1100	3000	100.0	177.4	76.4	835.3	297.9	82.8	0.0	1089.5	331.9	55.9	0.0
19 ≤ age < 31	208	208	800	2500	99.1	161.4	73.9	859.4	239.3	44.3	0.0	1107.7	295.5	14.1	0.0
31 ≤ age < 51	481	481	800	2500	99.2	145.0	68.8	790.2	257.8	56.8	0.0	1010.9	298.8	25.4	0.0
51 ≤ age < 71	418	418	800	2000	99.5	140.2	62.2	838.7	283.7	49.6	0.2	1055.4	305.2	20.6	0.6
age ≥ 71	105	105	1000	2000	100.0	129.1	52.4	917.2	275.0	66.2	0.2	1120.0	301.0	38.4	1.0

note: Shown for each age group are the number of individuals included in the analysis, dietary reference values for calcium (EAR and UL), percentage of individuals who consumed flour, mean flour intake taking into account all the individuals in the group and SD, calcium intake, adequacy of calcium intake, and calcium intake after fortifying flour with 156 mg of calcium per 100 g of flour.

EAR, estimated average requirement; UL, upper limit for calcium according to the dietary reference intakes of the Institute of Medicine Food and Nutrition Board.

**Table 4:** Results of the simulation for the Lao PDR: hypothetical changes in usual (adjusted) calcium intake after addition of 156 mg of calcium per 100 g of flour

					Flour consumption			Calcium intake				Calcium intake after fortification with 156 mg in 100 g flour			
Group (age in years)	N round 1	N round 2	EAR	UL	% population consuming flour	Mean flour intake (mg)	SD	Mean (mg)	SD	<EAR (%)	>UL (%)	Mean (mg)	SD	<EAR (%)	>UL (%)
**Children**															
0.5 ≤ age < 1	170	19	270	1500	28.8	2.2	5.8	328.8	398.6	62.3	2.0	332.2	401.1	61.7	2.0
1 ≤ age < 4	407	45	400	2500	62.4	9.5	15.6	364.1	257.9	66.8	0.0	378.6	258.8	64.6	0.0
4 ≤ age < 9	294	29	640	2500	74.5	16.7	23.4	214.1	104.6	99.5	0.0	238.8	106.2	99.5	0.0
**Nonpregnant women**															
9 ≤ age < 14	74	5	1100	3000	73.0	30.1	40.1	207.2	93.4	100.0	0.0	252.9	103.6	100.0	0.0
14 ≤ age < 19	38	4	1100	3000	55.3	30.5	40.1	207.7	166.8	99.5	0.0	257.2	165.3	99.6	0.0
19 ≤ age < 31	53	3	800	2500	34.0	22.6	44.1	263.5	109.9	99.8	0.0	295.7	115.0	99.8	0.0
31 ≤ age < 51	101	11	800	2500	34.7	18.4	30.7	250.6	105.4	99.8	0.0	278.0	107.5	99.8	0.0
51 ≤ age < 71	127	15	1000	2000	22.8	9.6	22.3	230.0	125.3	99.9	0.0	252.2	138.3	99.8	0.0
**Pregnant women**															
14 ≤ age < 19	23	3	1100	3000	39.1	18.4	29.3	229.6	103.1	100.0	0.0	291.0	101.8	100.0	0.0
19 ≤ age < 31	196	25	800	2500	33.7	18.4	35.8	313.7	185.5	97.6	0.0	342.2	146.3	98.9	0.0
31 ≤ age < 51	66	8	800	2500	37.9	20.3	42.7	300.6	167.1	98.3	0.0	332.9	167.1	98.1	0.0
**Male**															
19 ≤ age < 31	22	–	800	2500	31.8	17.8	29.1	212.0	62.5	100.0	0.0	240.5	67.5	100.0	0.0
31 ≤ age < 51	108	13	800	2500	19.4	11.0	28.8	435.6	381.3	89.7	0.5	421.0	291.3	91.3	0.1
51 ≤ age < 71	116	12	800	2000	11.2	4.8	15.3	276.1	137.8	99.3	0.0	292.0	146.1	99.0	0.0
age ≥ 71	21	3	1000	2000	23.8	5.4	15.5	342.4	222.0	98.1	0.1	354.9	237.7	97.6	0.2

note: Shown for each age group are the number of individuals included in the analysis, dietary reference values for calcium (EAR and UL), percentage of individuals who consumed flour, mean flour intake taking into account all the individuals in the group and SD, calcium intake, adequacy of calcium intake, and calcium intake after fortifying flour with 156 mg of calcium per 100 g of flour.

EAR, estimated average requirement; UL, the upper limit for calcium according to the dietary reference intakes of the Institute of Medicine Food and Nutrition Board.

**Table 5:** Results of the simulation for Uganda: hypothetical changes in usual (adjusted) calcium intake after addition of 156 mg of calcium per 100 g of flour

					Flour consumption			Calcium intake				Calcium intake after fortification with 156 mg in 100 g flour			
Group (age in years)	N round 1	N round 2	EAR	UL	% population consuming flour	Mean intake of flour (mg)	SD	Mean (mg)	SD	<EAR (%)	>UL (%)	Mean (mg)	SD	<EAR (%)	>UL (%)
**Nonpregnant women**															
19 ≤ age < 31	69	26	800	2500	72.5	231.3	204.6	462.7	283.7	89.8	0.0	783.2	404.0	58.1	0.2
31 ≤ age < 51	171	50	800	2500	77.8	249.7	208.7	363.3	163.2	98.1	0.0	739.1	261.6	62.9	0.0
51 ≤ age < 71	30	10	1000	2000	83.3	312.6	253.9	418.9	220.3	98.0	0.0	872.5	326.4	67.2	0.2
**Pregnant women**															
19 ≤ age < 31	172	53	800	2500	86.6	263.6	185.1	372.3	118.5	99.6	0.0	798.9	246.9	54.6	0.0
31 ≤ age < 51	123	38	800	2500	79.7	235.3	183.2	389.3	137.4	99.0	0.0	732.9	194.0	66.3	0.0

note: Shown for each age group are the number of individuals included in the analysis, dietary reference values for calcium (EAR and UL), percentage of individuals who consumed flour, mean flour intake taking into account all the individuals in the group and SD, calcium intake, adequacy of calcium intake, and calcium intake after fortifying flour with 156 mg of calcium per 100 g of flour.

EAR, estimated average requirement; UL, the upper limit for calcium according to the dietary reference intakes of the Institute of Medicine Food and Nutrition Board.

**Table 6:** Results of the simulation for the United States: hypothetical changes in usual (adjusted) calcium intake after addition of 156 mg of calcium per 100 g of flour

					Flour consumption			Calcium intake				Calcium intake after fortification with 156 mg in 100 g flour			
Group (age in years)	N round 1	N round 2	EAR	UL	% population consuming flour	Mean flour intake (mg)	SD	Mean (mg)	SD	<EAR (%)	>UL (%)	Mean (mg)	SD	<EAR (%)	>UL (%)
**Children**															
0.5 ≤ age < 1	126	99	270	1500	88.9	30.9	34.5	715.4	319.8	3.1	2.9	764.2	344.5	2.2	3.9
1 ≤ age < 4	564	457	400	2500	98.8	77.6	58.8	915.0	492.0	11.1	1.1	1036.1	530.2	7.1	1.8
4 ≤ age < 9	828	649	640	2500	98.4	119.4	70.0	952.3	487.6	28.9	0.7	1140.2	547.6	18.1	1.9
**Nonpregnant women**															
9 ≤ age < 14	417	330	1100	3000	98.3	127.0	77.0	919.1	547.9	69.2	0.4	1118.5	623.9	55.7	1.0
14 ≤ age < 19	383	311	1100	3000	97.4	123.1	79.7	826.8	520.9	75.4	0.3	1018.0	598.6	62.7	0.8
19 ≤ age < 31	511	411	800	2500	96.9	115.1	82.7	825.9	501.5	56.3	0.9	1005.0	572.0	42.1	1.9
31 ≤ age < 51	854	717	800	2500	97.2	109.3	77.0	837.7	471.8	54.6	0.6	1008.2	546.1	41.0	1.7
51 ≤ age < 71	845	740	1000	2000	96.0	96.3	69.1	775.7	436.4	74.9	1.6	926.2	496.5	62.7	3.5
age ≥ 71	381	327	1000	2000	96.3	82.3	56.2	731.7	418.3	78.6	1.3	861.3	456.0	68.6	2.3
**Pregnant women**															
19 ≤ age < 31	37	31	800	2500	97.3	151.0	106.2	999.0	542.3	40.5	1.2	1239.4	614.6	25.5	3.4
31 ≤ age < 51	26	20	800	2500	92.3	111.2	84.2	947.7	507.5	42.8	0.6	1123.3	590.4	31.8	1.8
**Male**															
9 ≤ age < 14	412	340	1100	3000	98.6	146.1	84.8	1039.3	650.0	62.2	1.3	1268.0	721.4	48.0	2.6
14 ≤ age < 19	374	304	1100	3000	99.2	158.9	101.9	1100.3	698.9	58.1	1.8	1348.7	810.5	44.7	4.2
19 ≤ age < 31	497	385	800	2500	94.6	169.2	128.9	1067.7	719.6	42.9	4.7	1329.0	845.5	30.1	9.4
31 ≤ age < 51	792	643	800	2500	95.7	154.1	109.8	1042.1	658.1	42.7	3.5	1279.1	773.9	30.2	7.5
51 ≤ age < 71	824	694	800	2000	95.8	130.0	93.7	939.1	581.7	47.9	5.8	1141.3	672.5	34.7	10.2
age ≥ 71	372	333	1000	2000	97.6	108.0	78.9	871.6	553.8	67.1	3.9	1040.9	605.7	55.4	7.4

note: Shown for each age group are the number of individuals included in the analysis, dietary reference values for calcium (EAR and UL), percentage of individuals who consumed flour, mean flour intake taking into account all the individuals in the group and SD, calcium intake, adequacy of calcium intake, and calcium intake after fortifying flour with 156 mg of calcium per 100 g of flour.

EAR, estimated average requirement; UL, the upper limit for calcium according to the dietary reference intakes of the Institute of Medicine Food and Nutrition Board.

**Table 7:** Results of the simulation for Zambia: hypothetical changes in usual (adjusted) calcium intake after addition of 156 mg of calcium per 100 g of flour

					Flour consumption			Calcium intake				Calcium intake after fortification with 156 mg in 100 g flour			
Group (age in years)	N round 1	N round 2	EAR	UL	% population consuming flour	Mean flour intake (mg)	SD	Mean (mg)	SD	<EAR (%)	>UL (%)	Mean (mg)	SD	<EAR (%)	>UL (%)
**Children**															
1 ≤ age < 4	322	280	400	2500	99.7	156.5	73.0	202.6	70.8	98.7	0.0	452.9	141.5	38.6	0.0
4 ≤ age < 9	132	121	640	2500	99.2	194.1	77.2	220.2	64.0	100.0	0.0	516.3	129.4	85.0	0.0
**Nonpregnant women**															
19 ≤ age < 31	73	65	800	2500	100.0	276.8	116.0	339.3	9.6	100.0	0.0	746.5	176.4	65.2	0.0
31 ≤ age < 51	72	61	800	2500	100.0	300.9	99.3	311.5	24.5	100.0	0.0	808.1	167.2	50.8	0.0
**Pregnant women**															
19 ≤ age < 31	126	108	800	2500	100.0	302.2	106.9	322.2	114.4	99.8	0.0	788.5	199.0	56.8	0.0
31 ≤ age < 51	60	56	800	2500	98.3	295.4	118.4	317.4	85.6	100.0	0.0	777.6	222.7	57.2	0.0

note: Shown for each age group are the number of individuals included in the analysis, dietary reference values for calcium (EAR and UL), percentage of individuals who consumed flour, mean flour intake taking into account all the individuals in the group and SD, calcium intake, adequacy of calcium intake, and calcium intake after fortifying flour with 156 mg of calcium per 100 g of flour.

EAR, estimated average requirement; UL, the upper level for calcium according to the dietary reference intakes of the Institute of Medicine Food and Nutrition Board.

The usual calcium intake was calculated using the information from repeated measures reported in the databases, except for Argentina, where the external variance provided by the IMAPP (the 2003–2008 NHANES coefficient of variance) was used, as the database did not have duplicate recalls or the number of cases with duplicate recalls was small. The results for the simulation exercise presented here include the mean calcium intake, prevalence of low calcium intake, and percentage of calcium intake above the UL for each age group available by country, before and after a hypothetical flour fortification process.

### Argentina

The mean and standard deviation (SD) daily calcium intake ranged from 609.3 (SD* = *348.0) to 752.2 (SD* = *323.4) mg in children, from 361.6 (SD* = *180.2) to 460.7 (SD* = *167.6) mg in NPW, and from 470.6 (SD* = *246.7) to 491.3 (SD* = *244.7) mg in PW. The prevalence of low calcium intake was 17.5% in children less than 1 year, 13.4% in children age 1 to less than 4 years, 46.2% in children 4 to less than 9 years, and 88% or higher in girls and women, including PW. None of the groups had individuals with calcium intake exceeding the recommended UL for calcium except for children between aged 6 months and less than 1 year where 1.8% exceeded the recommended UL (Table 1). The mean flour intake in adults ranged from 87.9 to 137.2 g per day, and it was mainly wheat.

After the simulation, the intake of flour fortified with 156 mg per 100 g, the prevalence of low calcium intake decreased from 17.5% to 14.4% in children less than 1 year (and a change from 1.5% to 1.8% of this population group above the UL), from 13.4% to 8.5% in children aged 1 to less than 4 years, and from 46.2% to 26.0% in children aged 4 to less than 9 years. The prevalence of low calcium intake was marginally reduced in NPW and PW (Table 1). None of the groups had more than 1.8% of the individuals with calcium intakes exceeding the UL. The original and after‐simulation calcium intake distributions are presented in Figure S1 (online only).

### Bangladesh

The mean and SD daily calcium intake in NPW ranged from 151.8 (SD* = *53.7) to 160.8 (SD* = *55.6) mg and from 143.2 (SD* = *80.3) to 150.2 (SD* = *58.8) mg in PW. The prevalence of low calcium intake was 100% in all age groups. None of the groups had individuals with calcium intake exceeding the recommended UL for calcium (Table 2). The percentage of the population with flour intake in Bangladesh and the amount of flour consumed was very low, so a flour fortification strategy in this population would not have an impact on calcium intake.

The original and after‐simulation calcium intake distributions are presented in Figure S2 (online only).

### Italy

The mean and SD of daily calcium intake ranged from 600.0 (SD* = *147.3) to 728.6 (SD* = *180.5) mg in children, from 822.0 (SD* = *267.6) to 1009.1 (SD* = *181.2) mg in PW, from 716.4 (SD* = *237.5) to 819.5 (SD* = *187.0) mg in girls and NPW, and from 790.2 (SD* = *257.8) to 917.2 (SD* = *275.0) mg in boys and men. The prevalence of low calcium intake ranged from 1.7% to 37.2% in children, from 13.1% to 51.0% in PW, from 65.7% to 92.3% in girls and NPW, and from 44.3% to 82.8% in boys and men. None of the groups had more than 1.0% of the individuals with calcium intakes exceeding the recommended UL for calcium (Table 3). The mean flour intake in adults ranged from 104.0 to 177.4 g per day, and it was mainly wheat.

After simulating the intake of flour fortified with 156 mg per 100 g, the prevalence of low calcium intake decreased from 1.7% to 1% in children less than 1 year, from 1.9% to 0.3% in children aged 1 to less than 4 years, and from 37.2% to 12.6% in children aged 4 to less than 9 years. The prevalence of low calcium intake was reduced from 88.9% to 69.1% in girls from 9 to less than 14 and from 92.3% to 64.8% in girls from 14 to less than 19.

The prevalence of low calcium intake was reduced from 68.4% to 37.3% in the group of NPW aged 19 to less than 31 years, from 65.7% to 36.6% in the group of NPW aged 31 to less than 51 years, from 84.7% to 66.6% in the group of NPW aged 51 to less than 71 years, from 13.1% to 0.6% in PW aged 19 to less than 31 years, and from 51.0% to 22.9% in PW 31 to less than 51 years (Table 3). None of the groups had more than 1% of the individuals with calcium intakes exceeding the UL.

The prevalence of low calcium intake was reduced from around 80% to 55% in boys aged 9 to less than 19, from 44.3% to 14.1% in men aged 19 to less than 31, from 56.8% to 25.4% in men aged 31 to less than 51, from 49.6% to 20.6% in men aged 51 to less than 71, and from 66.2% to 38.4% in men aged over 71.

The original and after‐simulation calcium intake distributions are presented in Figure S3 (online only).

### The Lao PDR

The mean and SD daily calcium intake ranged from 214.1 (SD* = *104.6) to 364.1 (SD* = *257.9) mg in children, from 207.2 (SD* = *93.4) to 263.5 (SD* = *109.9) mg in girls and NPW, from 229.6 (SD* = *103.1) to 313.7 (SD* = *185.5) mg in PW, and from 212.0 (SD* = *62.5) to 435.6 (SD* = *381.3) mg in men. The prevalence of low calcium intake ranged from 62.3% to 99.5% in children, and it was close to 100% in the rest of the groups, and none of them had calcium intake higher than 2% of the UL (Table 4).

The percentage of the population consuming flour and the amount of flour intake was very low; therefore, this strategy shows very little impact on the reduction of low calcium intake (Table 4).

The original and after‐simulation calcium intake distributions are presented in Figure S4 (online only).

### Uganda

The mean and SD daily calcium intake ranged from 363.3 (SD* = *163.2) to 462.7 (SD* = *283.7) mg in NPW and from 372.3 (SD* = *118.5) to 389.3 (SD* = *137.4) mg in PW. The prevalence of low calcium intake was 90% or higher. None of the population age groups had calcium intake exceeding the recommended UL for calcium (Table 5). The mean flour intake in adults ranged from 231.3 to 312.6 g per day, and it was mainly cassava and maize flour.

After simulating the intake of flour fortified with 156 mg of calcium per 100 mg, the prevalence of low calcium intake decreased from 89.8% to 58.1% in the group of NPW aged 19 to less than 31 years, from 98.1% to 62.9% in the group of NPW aged 31 to less than 51 years, from 98.0% to 67.2% in the group of NPW aged 51 to less than 71 years, from 99.6% to 54.6% in PW aged 19 to less than 31 years, and from 99.0% to 66.3% in PW 31 to less than 51 years (Table 5). None of the groups had more than 0.2% of the individuals with calcium intake exceeding the recommended UL for calcium.

The original and after‐simulation calcium intake distributions are presented in Figure S5 (online only).

### The United States

The mean and SD daily calcium intake ranged from 715.4 (SD* = *319.8) to 952.3 (SD* = *487.6) mg in children, from 731.7 (SD* = *418.3) to 919.1 (SD* = *547.9) mg in girls and NPW, from 947.7 (SD* = *507.5) to 999.0 (SD* = *542.3) mg in PW, and from 871.6 (SD* = *553.8) to 1100.3 mg (SD* = *698.9) mg in boys and men. The prevalence of low calcium intake ranged from 3.1% to 28.9% in children, from 54.6% to 78.6% in girls and NPW, from 40.5% to 42.8% in PW, and from 42.7% to 67.1% in boys and men. Children and women did not exceed the calcium intake UL in more than 2.9%, whereas around 3.5–5.8% of the individuals exceeded the UL in the group of men older than 19 years (Table 6). The mean flour intake in adults ranged from 82.3 to 169.2 g per day, and it was mainly wheat.

After simulating the intake of flour fortified with 156 mg per 100 g, the prevalence of low calcium intake decreased from 3.1% to 2.2% in children less than 1 year, from 11.1% to 7.1% in children aged 1 to less than 4 years, and from 28.9% to 18.1% in children aged 4 to less than 9 years. The prevalence of low calcium intake was reduced from 69.2% to 55.7% in girls from 9 to less than 14 years and from 75.4% to 62.7% in girls from 14 to less than 19 years.

The prevalence of low calcium intake was reduced to 42.1% in the group of NPW aged 19 to less than 31 years, 41.0% in the group of NPW aged 31 to less than 51 years; 62.7% in the group of NPW aged 51 to less than 71 years; 68.6% in the group of NPW aged over 71; 25.5% in PW aged 19 to less than 31 years; and 31.8% in PW 31 to less than 51 years old (Table 6). None of the groups had more than 3.9% of the individuals with calcium intakes exceeding the UL.

The prevalence of low calcium intake was reduced from 62.2% to around 48.0% in boys aged 9 to less than 14, from 58.1% to 44.7% in boys aged 14 to less than 19; from around 43% to around 30% in men aged 19 to less than 51; from 47.9% to 34.7% in men aged 51 to less than 71; and from 67.1% to 55.4% in men aged over 71. In the group of men aged 19 to over 71, between 7.4% and 10.2% of them exceeded the UL (Table 6).

The original and after‐simulation calcium intake distributions are presented in Figure S6 (online only).

### Zambia

The mean and SD daily calcium intake ranged from 202.6 (SD* = *70.8) to 220.2 mg (SD* = *64.0) in children, from 311.5 (SD* = *24.5) to 339.3 mg (SD* = *9.6) in NPW, and from 317.4 (SD* = *85.6) to 322.2 mg (SD* = *114.4) in PW. The prevalence of low calcium intake was 98% or higher. All of the groups had 0.0% of the individuals with calcium intakes exceeding the recommended UL for calcium (Table 7). The mean flour intake in adults ranged from 276.8 to 302.2 g per day, and it was mainly maize flour.

After the simulation, the intake of flour fortified with 156 mg per 100 g, the prevalence of low calcium intake decreased from 98.7% to 38.6% in children 1 to less than 4 years; from 100.0% to 85% in children 4 to less than 9 years; and from 100.0% to between 50.8% and 65.2% in NPW and PW from 19 to less than 51 (Table 7). All of the groups had 0.0% of the individuals with calcium intakes exceeding the UL.

The original and after‐simulation calcium intake distributions are presented in Figure S7 (online only).

**Figure 1:**
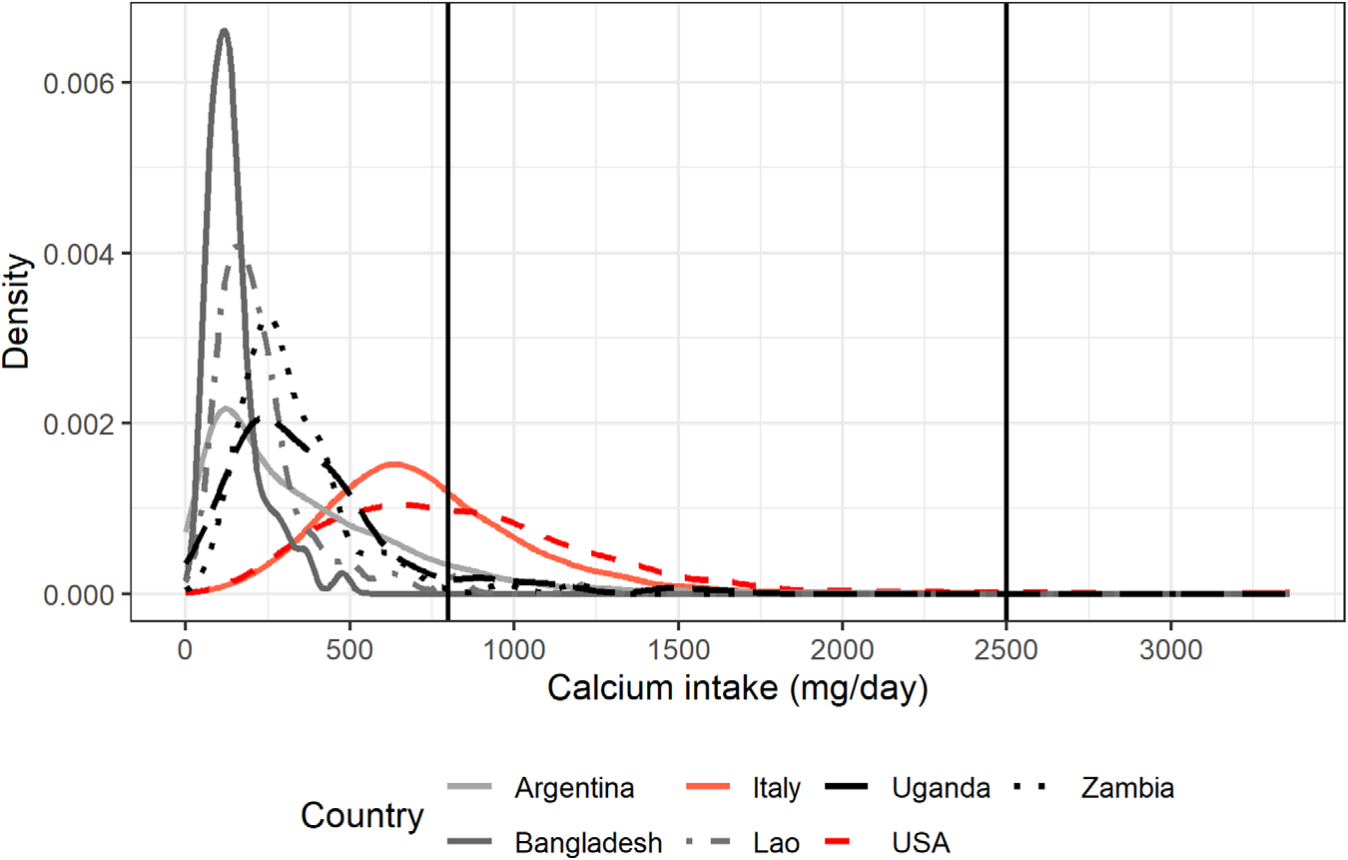
Distribution of mg of adjusted daily calcium intake of NPW aged between 31 and 50.9 years for Argentina, Bangladesh, Italy, the Lao PDR, Uganda, the United States, and Zambia.

**Figure 2:**
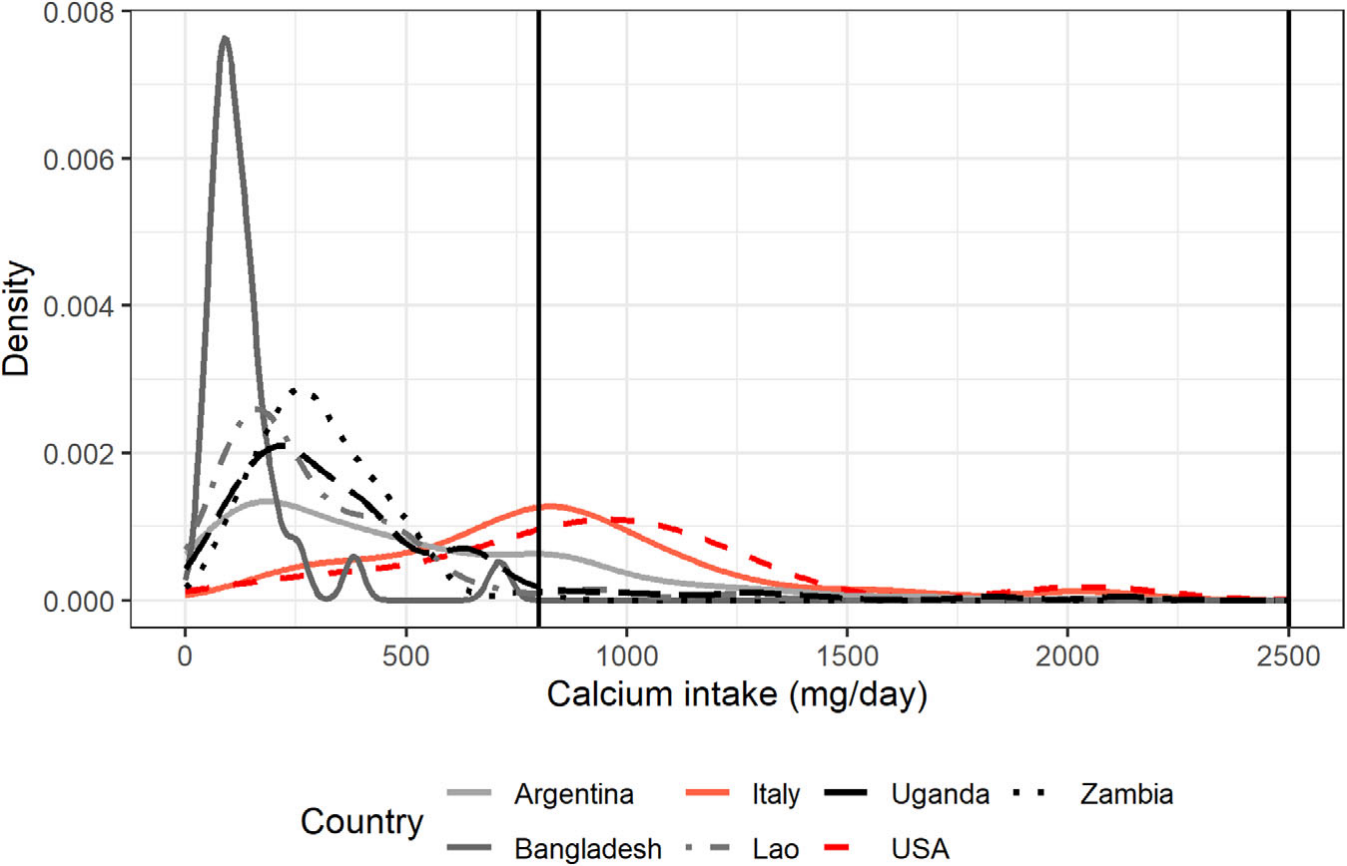
Distribution of mg of adjusted daily calcium intake of PW aged between 31 and 50.9 years for Argentina, Bangladesh, Italy, the Lao PDR, Uganda, the United States, and Zambia.

**Figure 3:**
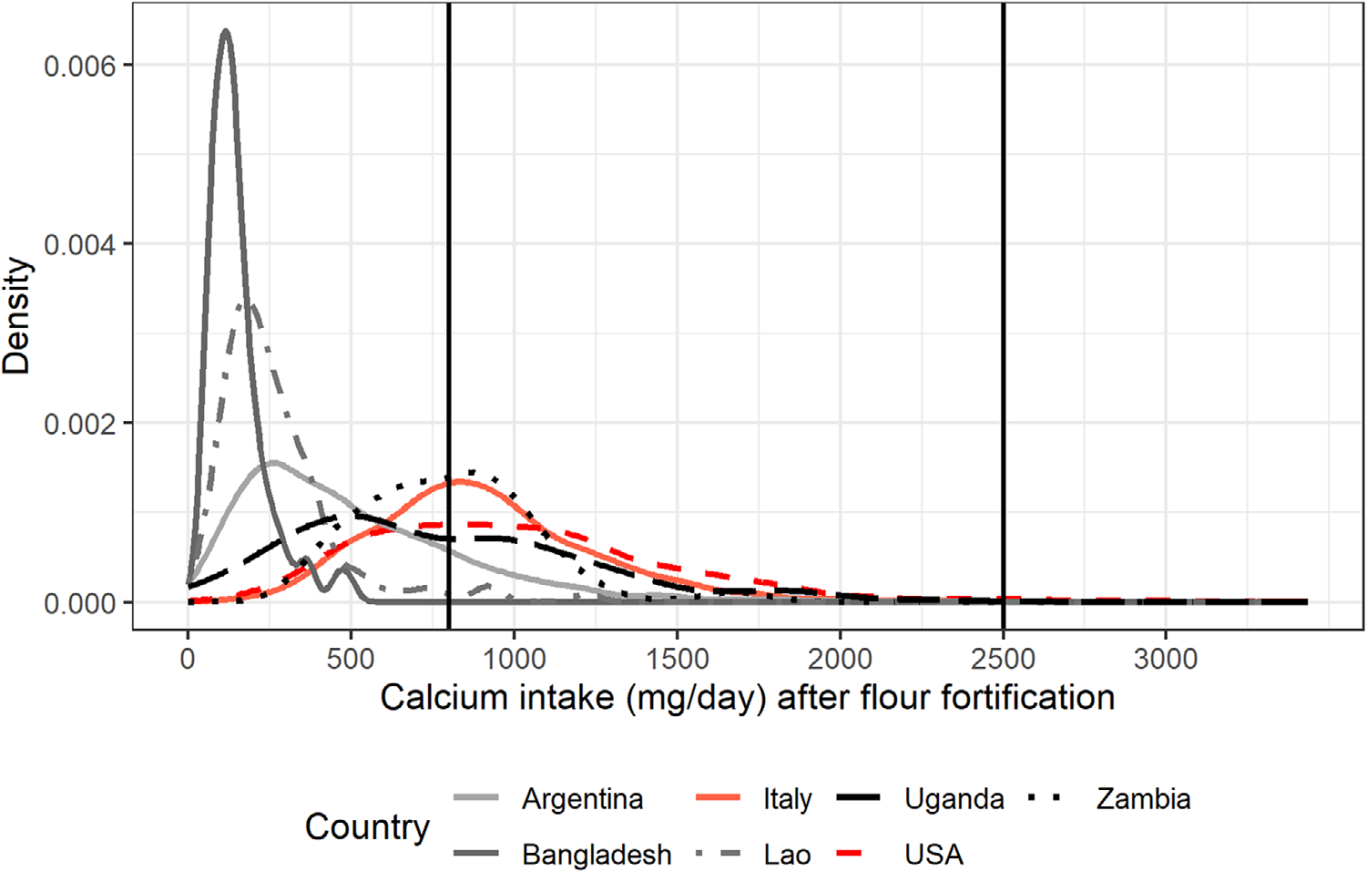
Distribution of mg of adjusted daily calcium intake of NPW aged between 31 and 50.9 years after simulating fortification of flour with 156 mg of calcium per 100 g of flour for Argentina, Bangladesh, Italy, the Lao PDR, Uganda, the United States, and Zambia.

**Figure 4:**
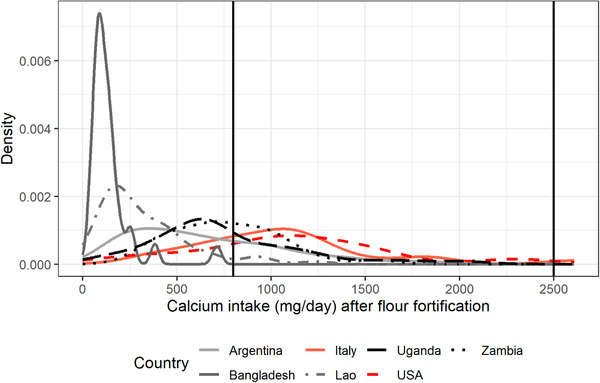
Distribution of mg of adjusted daily calcium intake of PW aged between 31 and 50.9 years after simulating fortification of flour with 156 mg of calcium per 100 g of flour for Argentina, Bangladesh, Italy, the Lao PDR, Uganda, the United States, and Zambia.

Finally, as an example, Figures 1 and 2 present the distribution of mg of adjusted daily calcium intake of NPW and PW aged between 31 and 50.9 years, respectively, for Argentina, Bangladesh, Italy, the Lao PDR, Uganda, the United States, and Zambia. Figures 3 and 4 present the distribution of mg of daily calcium intake after simulating fortification of flour with 156 mg of calcium per 100 g of flour for the same groups. Figures 3 and 4 show that after flour fortification, most of the populations in HICs had calcium intakes between 800 (EAR) and 2500 (UL) mg of calcium a day, which are the limits considered safe for calcium, whereas LMICs had most of their populations under 800 mg of calcium a day.

## Discussion

This simulation exercise shows that calcium flour fortification strategy with 156 mg of calcium per 100 g of flour could improve the calcium intake in those populations where flour is consumed by most of the population and that in this theoretical model, the impact of the fortification strategy would vary according to the amount and distribution of flour consumed by the population. The simulation exercise shows that flour fortification would decrease the proportion of individuals with inadequate calcium intake in Argentina, Italy, Uganda, and Zambia. On the other hand, according to the model presented in this study, the strategy would not be suitable for Bangladesh or the Lao PDR, where the percentage of the population consuming flour is very low. For these countries, fortification strategies should probably focus on food vehicles more frequently consumed by the target populations. Similarly, this strategy would not be appropriate for countries where calcium intake is only inadequate in some population groups and where flour intake is high and widespread, such as in the United States.

We selected the UK level of fortification (156 mg of calcium per 100 g of flour) as it was the only mandatory flour fortification example currently available. This is just an exercise, and we reinforce that the application of this strategy should be replicated with the most recent dietary intake data for the aimed population. Furthermore, the technical feasibility analysis of calcium fortification of other types of flours and with other fortification levels besides white flour should be performed, as well as stability and organoleptic test of products prepared with fortified flours. Although only white flour is fortified in the United Kingdom, we applied it to any type of flour consumed. Also, there is a need to explore other vehicles to accomplish the desired target in countries like Bangladesh and the Lao PDR, where flour is not commonly consumed.

The model and analysis presented here have some limitations. This study is based on simulation with theoretical assumptions. The analysis was performed with available databases where adult men information was very scarce, and thus, it was impossible to evaluate the impact and risk in this group in all countries. Also, the database from Argentina was adjusted with external data as the survey did not have repeated measurements. Furthermore, some databases are old, and flour and calcium intake could have changed in these populations. Lastly, the fortification level was selected according to the current UK wheat fortification, and, therefore, there is a need to explore not only the calcium fortification level needed but also how this fortification level would affect the different stages of flour fortification, including but not limited to, flour production, cost, interactions with food matrix, products prepared from fortified flour and with the whole diet, and acceptability and access. Another limitation is that we used available data on flour content of foods, a proper analysis would require using the exact chemical composition of foods consumed in each of the countries evaluated.

In this exercise, we selected the current mandatory calcium fortification level for white flour used in the United Kingdom; however, we applied it to all types of flour. This selection would probably overestimate the effect of the flour fortification strategy; however, we selected this scenario to assess the risk of calcium intake excess measured by the percentage of people above the UL. Assessments of the technical feasibility of adding this amount to other flours besides white flour should be done.

The fortification level of 156 mg of calcium per 100 g of flour would probably be too high for the UK if all types of flour were under the mandatory flour fortification regulations; however, it does not seem to be high for the LMIC populations we include in this exercise, as they all improve their diet and none showed the risk of excess (over the UL).

Further analysis would require to explore the distribution of each type of flour intake so as to identify if there is a type of flour that would eventually have more impact on the most vulnerable population and to explore the interaction with other fortification micronutrients already approved in flour. Besides, the cost‐effectiveness of this strategy should be evaluated for each country; analysis in the UK assessed that stopping mandatory calcium fortification would increase social care costs by £3.06 million per year and £22.39 million per year in the National Health Service.^29^

There is limited information on the effect of calcium fortification strategies on clinical outcomes. Most of the evidence comes from calcium supplementation trials, where there have been concerns related to the effects of calcium supplements on iron absorption. On the basis of short‐term studies reporting a reduction in iron absorption of up to 55% depending on the dose, type of salt used, time of supplementation, and the presence of heme or nonheme iron in the food.^37^ However, evidence from long‐term calcium supplementation trials shows no effect.^10^ Furthermore, following this evidence, concomitant supplementation of iron and folic acid and calcium has been recommended for PW to improve supplementation adherence.^38^

## Conclusion

In view of the benefits of adequate calcium intake and the low intake of calcium in populations of LMICs, strategies to increase calcium intake are needed. While strategies to improve food habits and access to health and nutritional facilities to obtain calcium supplements are put in place, flour fortification with calcium could be considered as a strategy to contribute to increasing calcium intake at the population level. On the basis of the magnitude of calcium inadequacy and with more updated information on calcium and flour intake, each country should select a strategy or combination of strategies that better suits its population’s needs. This study shows that in some LMICs, flour fortification would, in theory, increase calcium intake without putting the population at risk of excess of calcium intake. However, in two LMICs, flour intake was so low that the impact on calcium intake was negligible, and fortification of other widely consumed foods should be considered. Results shown in this study are limited to the countries selected, but it can be hypothesized that similar results would be obtained in other LMICs. The analysis performed in this study is required in any country with the most updated information before implementing a fortification strategy.

## Author contributions

G.C. and A.P.B. were involved with conceptualization. L.G. and G.C. performed formal analysis. I.R. and S.P. carried out investigation. I.R., S.P., and G.C. were involved with data curation. G.C. and J.B. wrote the article. All authors wrote, reviewed, and edited the article. All authors have read and agreed to the published version of the article.

## Competing interests

The authors declare no competing interests.

## Supporting information


**Figure S1**. Argentina: distribution of calcium intake pre‐ and post‐flour fortification simulation.
**Figure S2**. Bangladesh: distribution of calcium intake pre‐ and post‐flour fortification simulation.
**Figure S3**. Italy: distribution of calcium intake pre‐ and post‐flour fortification simulation.
**Figure S4**. The Lao PDR: distribution of calcium intake pre‐ and post‐flour fortification simulation.
**Figure S5**. Uganda: distribution of calcium intake pre‐ and post‐flour fortification simulation.
**Figure S6**. The United States: distribution of calcium intake pre‐ and post‐flour fortification simulation.
**Figure S7**. Zambia: distribution of calcium intake pre‐ and post‐flour fortification simulation.
