# Supplementary material for: Impact of flour fortification with calcium on calcium intake: a simulation study in seven countries
Source: Ann N Y Acad Sci. 2021 Jan 11;1493(1):59–74. doi: 10.1111/nyas.14550 (PMC9290501; doi:10.1111/nyas.14550)
Supplement: Supplementary file 1 — Figure S1. Argentina: distribution of calcium intake pre‐ and post‐flour fortification simulation. Figure S2. Bangladesh: distribution of calcium intake pre‐ and post‐flour fortification simulation. Figure S3. Italy: distribution of calcium intake pre‐ and post‐flour fortification simulation. Figure S4. The Lao PDR: distribution of calcium intake pre‐ and post‐flour fortification simulation. Figure S5. Uganda: distribution of calcium intake pre‐ and post‐flour fortification simulation. Figure S6. The United States: distribution of calcium intake pre‐ and post‐flour fortification simulation. Figure S7. Zambia: distribution of calcium intake pre‐ and post‐flour fortification simulation. [file NYAS-1493-59-s001.zip › Supporting Information_annals-2000-338.R1.docx]

**Supporting information:**

**Figure S1.** Argentina: distribution of calcium intake pre- and post-flour fortification simulation. Black line: baseline calcium mean intake. Dashed line: estimated average requirement (EAR). Dotted line: upper limit (UL) of the required intake. Black density distribution: distribution of the baseline calcium intake. Red density distribution: distribution of the simulated calcium intake after flour fortification with 156 mg/L.

**Figure S2.** Bangladesh: distribution of calcium intake pre- and post-flour fortification simulation. Black line: baseline calcium mean intake. Dashed line: estimated average requirement (EAR). Dotted line: upper limit (UL) of the required intake. Black density distribution: distribution of the baseline calcium intake. Red Density distribution: distribution of the simulated calcium intake after flour fortification with 156 mg/L.

**Figure S3.** Italy: distribution of calcium intake pre- and post-flour fortification simulation. Black line: baseline calcium mean intake. Dashed line: estimated average requirement (EAR). Dotted line: upper limit (UL) of the required intake. Black density distribution: distribution of the baseline calcium intake. Red density distribution: distribution of the simulated calcium intake after flour fortification with 156 mg/L.

**Figure S4.** Lao PDR: distribution of calcium intake pre- and post-flour fortification simulation. Black line: baseline calcium mean intake. Dashed line: estimated average requirement (EAR). Dotted line: upper limit (UL) of the required intake. Black density distribution: distribution of the baseline calcium intake. Red density distribution: distribution of the simulated calcium intake after flour fortification with 156 mg/L.

**Figure S5.** Uganda: distribution of calcium intake pre- and post-flour fortification simulation. Black line: baseline calcium mean intake. Dashed line: estimated average requirement (EAR). Dotted line: upper limit (UL) of the required intake. Black density distribution: distribution of the baseline calcium intake. Red density distribution: distribution of the simulated calcium intake flour fortification with 156 mg/L.

**Figure S6.** USA: distribution of calcium intake pre- and post-flour fortification simulation. Black line: baseline calcium mean intake. Dashed line: estimated average requirement (EAR). Dotted line: upper limit (UL) of the required intake. Black density distribution: distribution of the baseline calcium intake. Red density distribution: distribution of the simulated calcium intake after flour fortification with 156 mg/L.

**Figure S7.** Zambia: distribution of calcium intake pre- and post-flour fortification simulation. Black line: baseline calcium mean intake. Dashed line: estimated average requirement (EAR). Dotted line: upper limit (UL) of the required intake. Black density distribution: distribution of the baseline calcium intake. Red density distribution: distribution of the simulated calcium intake after flour fortification with 156 mg/L.
