# Supplementary figures and images for: Impact of flour fortification with calcium on calcium intake: a simulation study in seven countries
Source: Ann N Y Acad Sci. 2021 Jan 11;1493(1):59–74. doi: 10.1111/nyas.14550 (PMC9290501; doi:10.1111/nyas.14550)

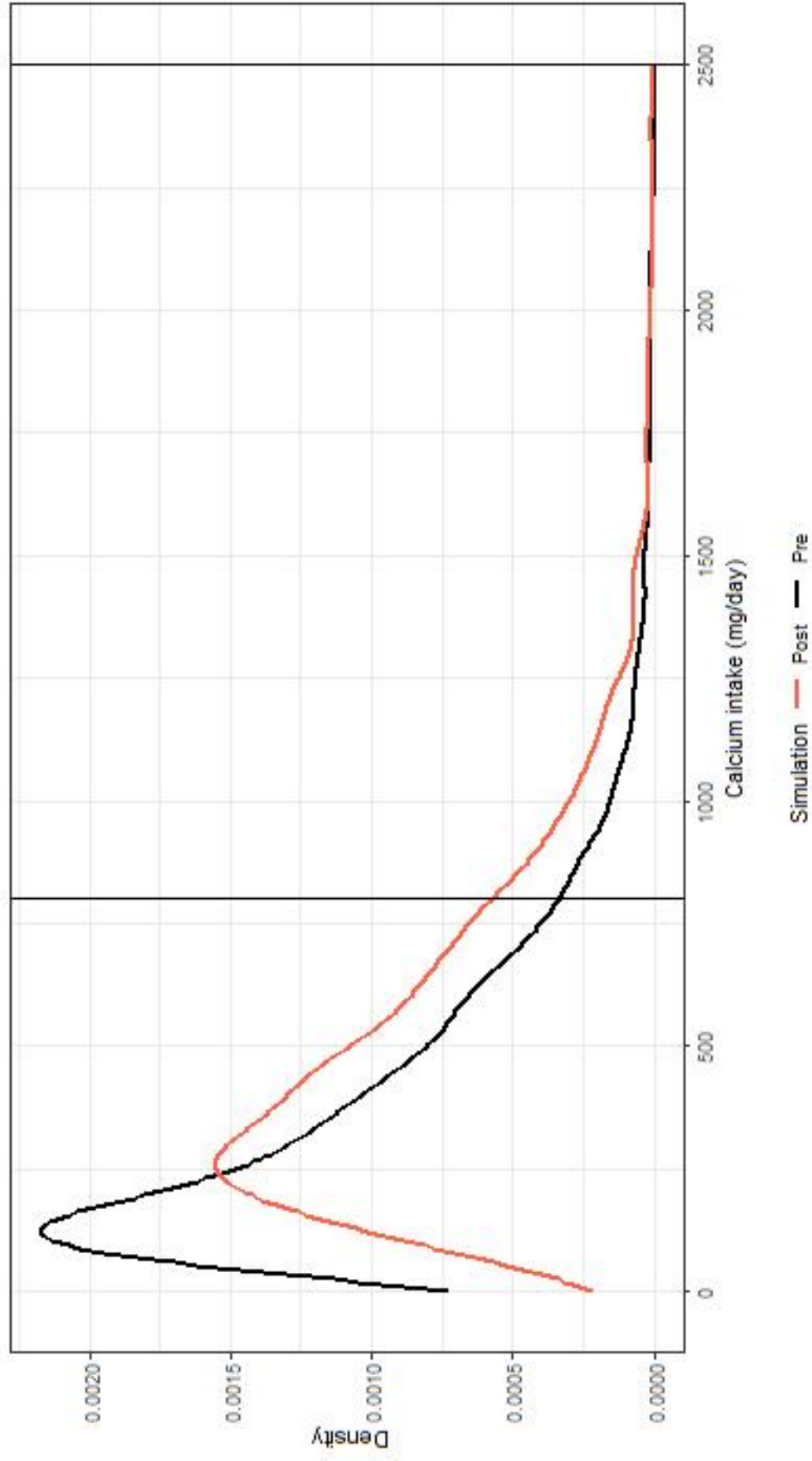

Supplement: Supplementary file 1 — Figure S1. Argentina: distribution of calcium intake pre‐ and post‐flour fortification simulation. Figure S2. Bangladesh: distribution of calcium intake pre‐ and post‐flour fortification simulation. Figure S3. Italy: distribution of calcium intake pre‐ and post‐flour fortification simulation. Figure S4. The Lao PDR: distribution of calcium intake pre‐ and post‐flour fortification simulation. Figure S5. Uganda: distribution of calcium intake pre‐ and post‐flour fortification simulation. Figure S6. The United States: distribution of calcium intake pre‐ and post‐flour fortification simulation. Figure S7. Zambia: distribution of calcium intake pre‐ and post‐flour fortification simulation. [file NYAS-1493-59-s001.zip › Figure S1 - Argentina Simulation of Flour Fortification.pdf]

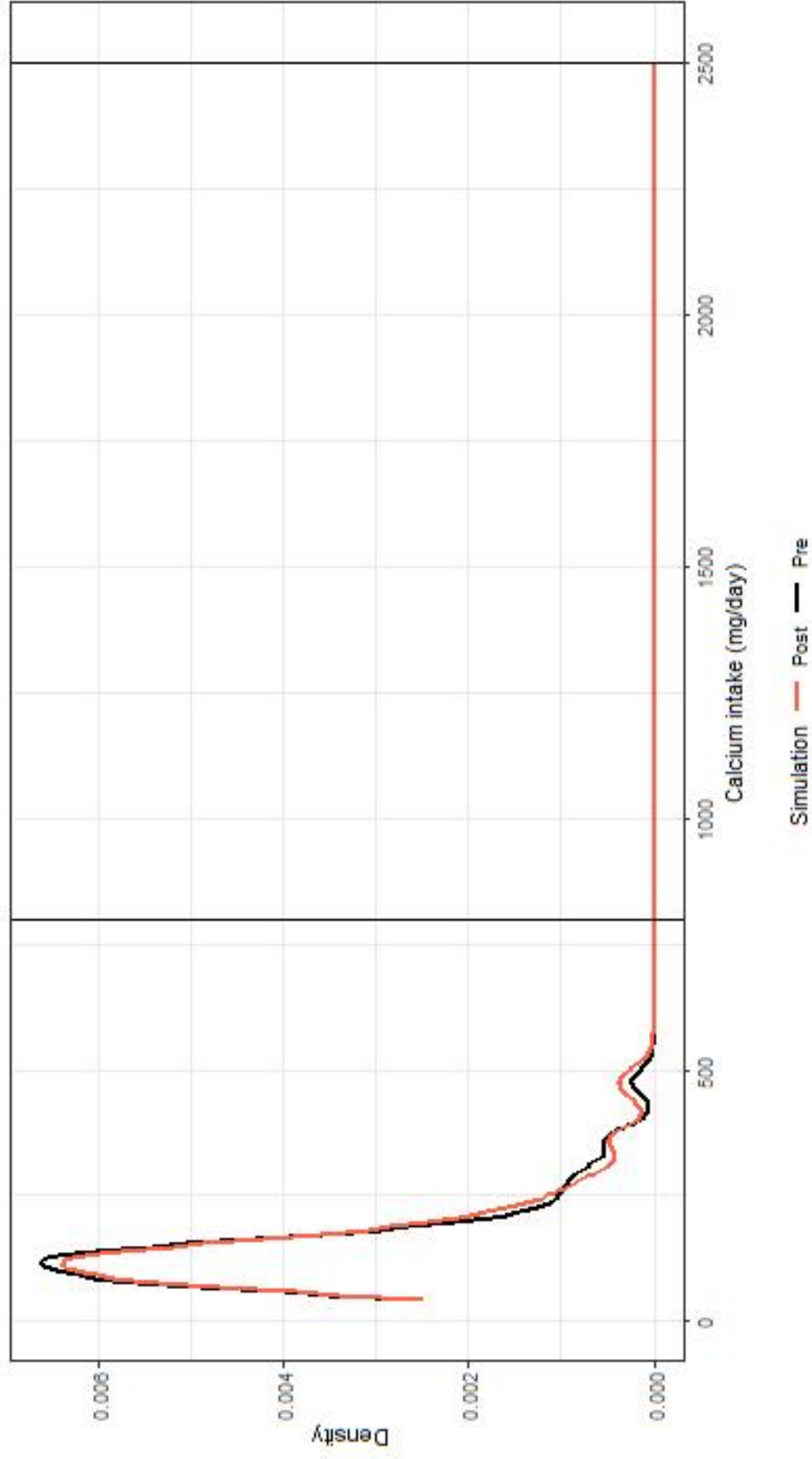

Supplement: Supplementary file 1 — Figure S1. Argentina: distribution of calcium intake pre‐ and post‐flour fortification simulation. Figure S2. Bangladesh: distribution of calcium intake pre‐ and post‐flour fortification simulation. Figure S3. Italy: distribution of calcium intake pre‐ and post‐flour fortification simulation. Figure S4. The Lao PDR: distribution of calcium intake pre‐ and post‐flour fortification simulation. Figure S5. Uganda: distribution of calcium intake pre‐ and post‐flour fortification simulation. Figure S6. The United States: distribution of calcium intake pre‐ and post‐flour fortification simulation. Figure S7. Zambia: distribution of calcium intake pre‐ and post‐flour fortification simulation. [file NYAS-1493-59-s001.zip › Figure S2 - Bangladesh Simulation of Flour Fortification.pdf]

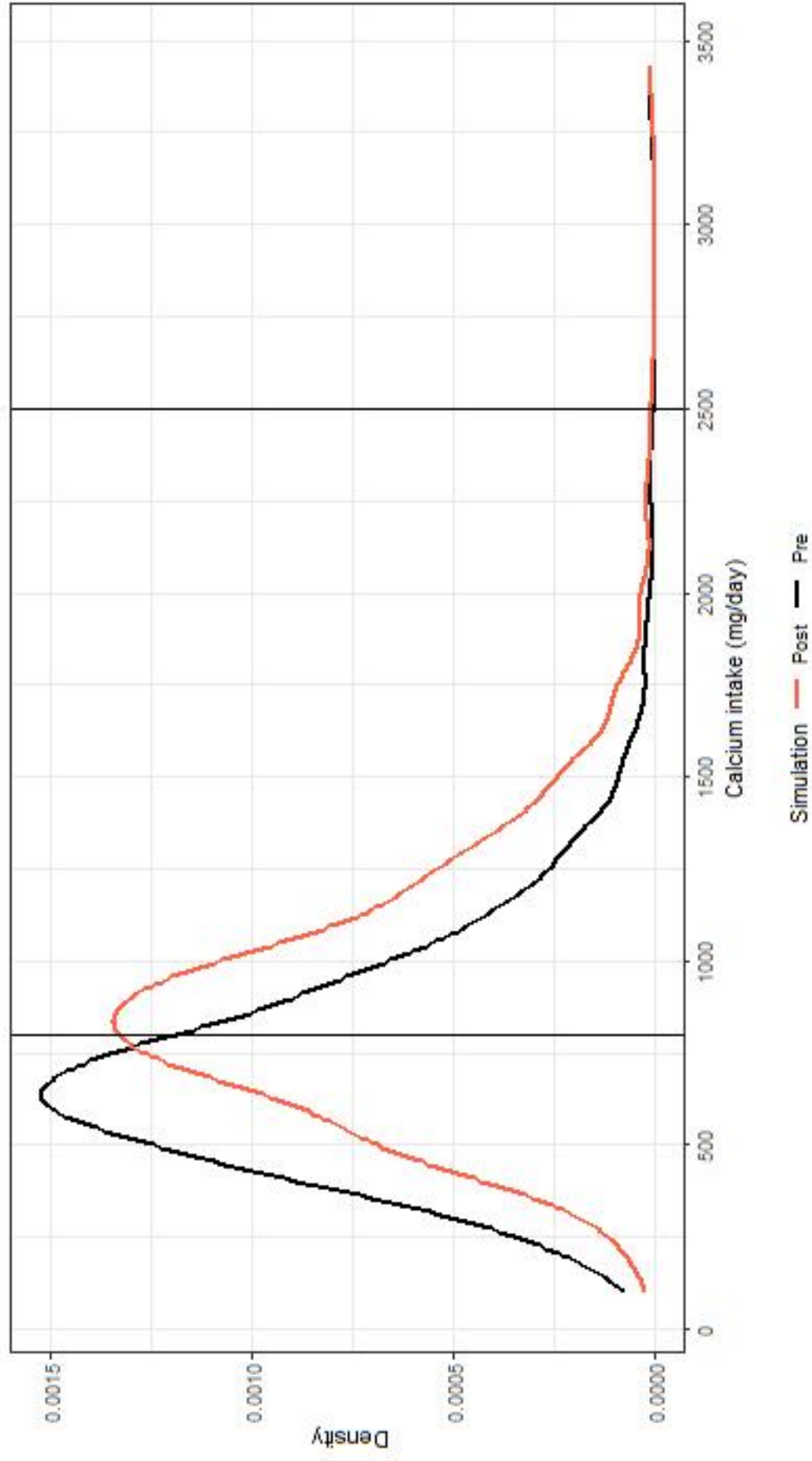

Supplement: Supplementary file 1 — Figure S1. Argentina: distribution of calcium intake pre‐ and post‐flour fortification simulation. Figure S2. Bangladesh: distribution of calcium intake pre‐ and post‐flour fortification simulation. Figure S3. Italy: distribution of calcium intake pre‐ and post‐flour fortification simulation. Figure S4. The Lao PDR: distribution of calcium intake pre‐ and post‐flour fortification simulation. Figure S5. Uganda: distribution of calcium intake pre‐ and post‐flour fortification simulation. Figure S6. The United States: distribution of calcium intake pre‐ and post‐flour fortification simulation. Figure S7. Zambia: distribution of calcium intake pre‐ and post‐flour fortification simulation. [file NYAS-1493-59-s001.zip › Figure S3 - Italy Simulation of Flour Fortification.pdf]

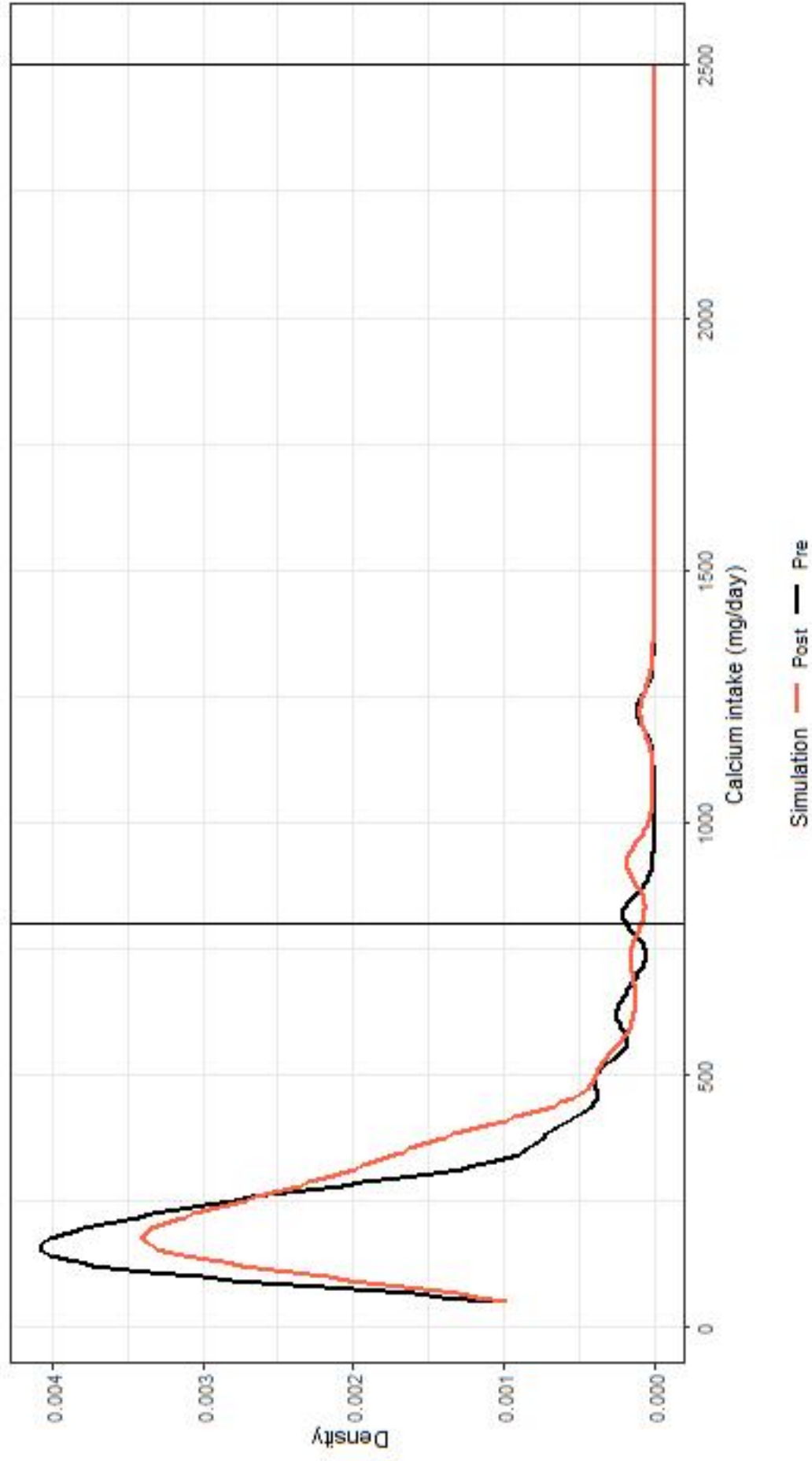

Supplement: Supplementary file 1 — Figure S1. Argentina: distribution of calcium intake pre‐ and post‐flour fortification simulation. Figure S2. Bangladesh: distribution of calcium intake pre‐ and post‐flour fortification simulation. Figure S3. Italy: distribution of calcium intake pre‐ and post‐flour fortification simulation. Figure S4. The Lao PDR: distribution of calcium intake pre‐ and post‐flour fortification simulation. Figure S5. Uganda: distribution of calcium intake pre‐ and post‐flour fortification simulation. Figure S6. The United States: distribution of calcium intake pre‐ and post‐flour fortification simulation. Figure S7. Zambia: distribution of calcium intake pre‐ and post‐flour fortification simulation. [file NYAS-1493-59-s001.zip › Figure S4 - Lao Simulation of Flour Fortification.pdf]

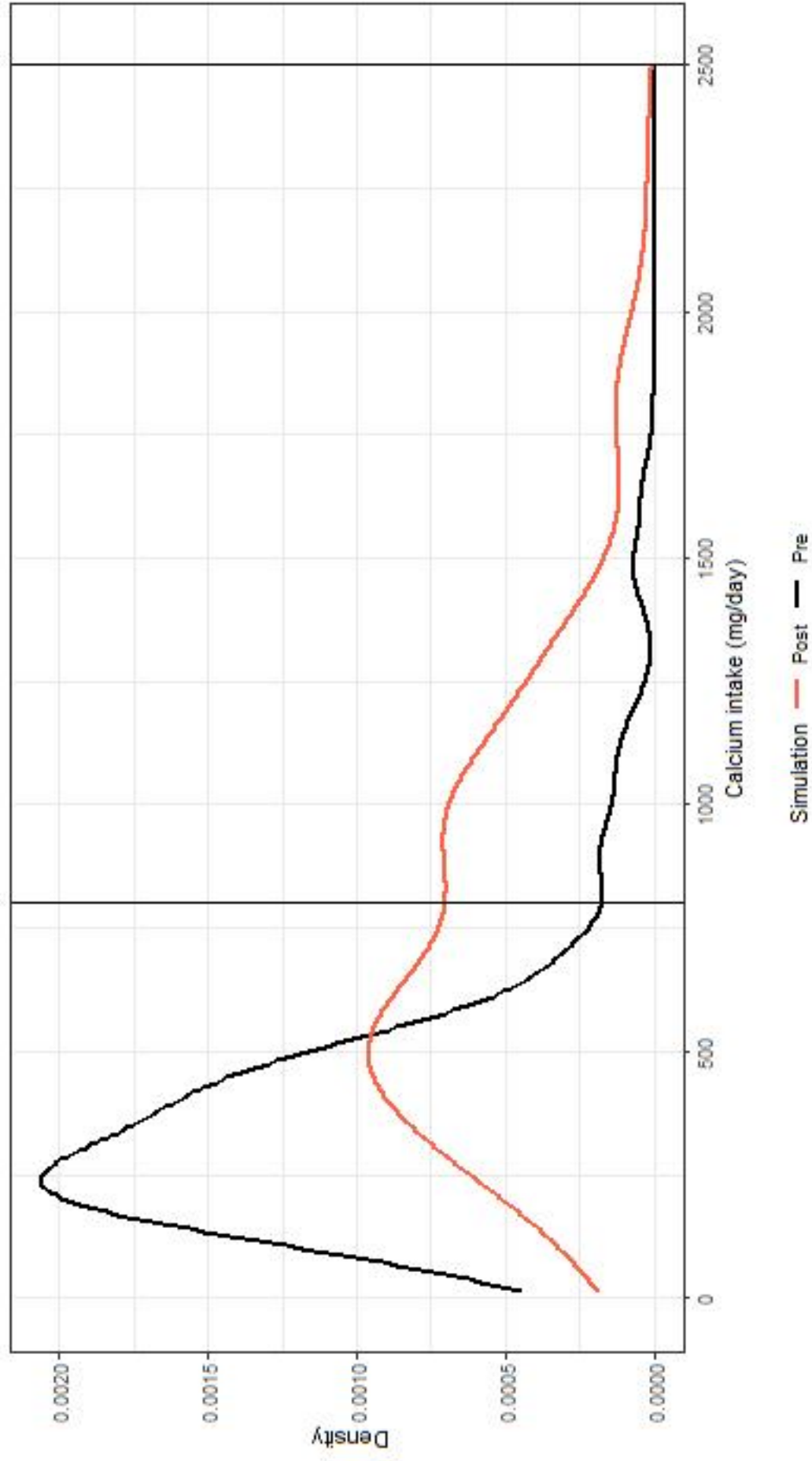

Supplement: Supplementary file 1 — Figure S1. Argentina: distribution of calcium intake pre‐ and post‐flour fortification simulation. Figure S2. Bangladesh: distribution of calcium intake pre‐ and post‐flour fortification simulation. Figure S3. Italy: distribution of calcium intake pre‐ and post‐flour fortification simulation. Figure S4. The Lao PDR: distribution of calcium intake pre‐ and post‐flour fortification simulation. Figure S5. Uganda: distribution of calcium intake pre‐ and post‐flour fortification simulation. Figure S6. The United States: distribution of calcium intake pre‐ and post‐flour fortification simulation. Figure S7. Zambia: distribution of calcium intake pre‐ and post‐flour fortification simulation. [file NYAS-1493-59-s001.zip › Figure S5 - Uganda Simulation of Flour Fortification.pdf]

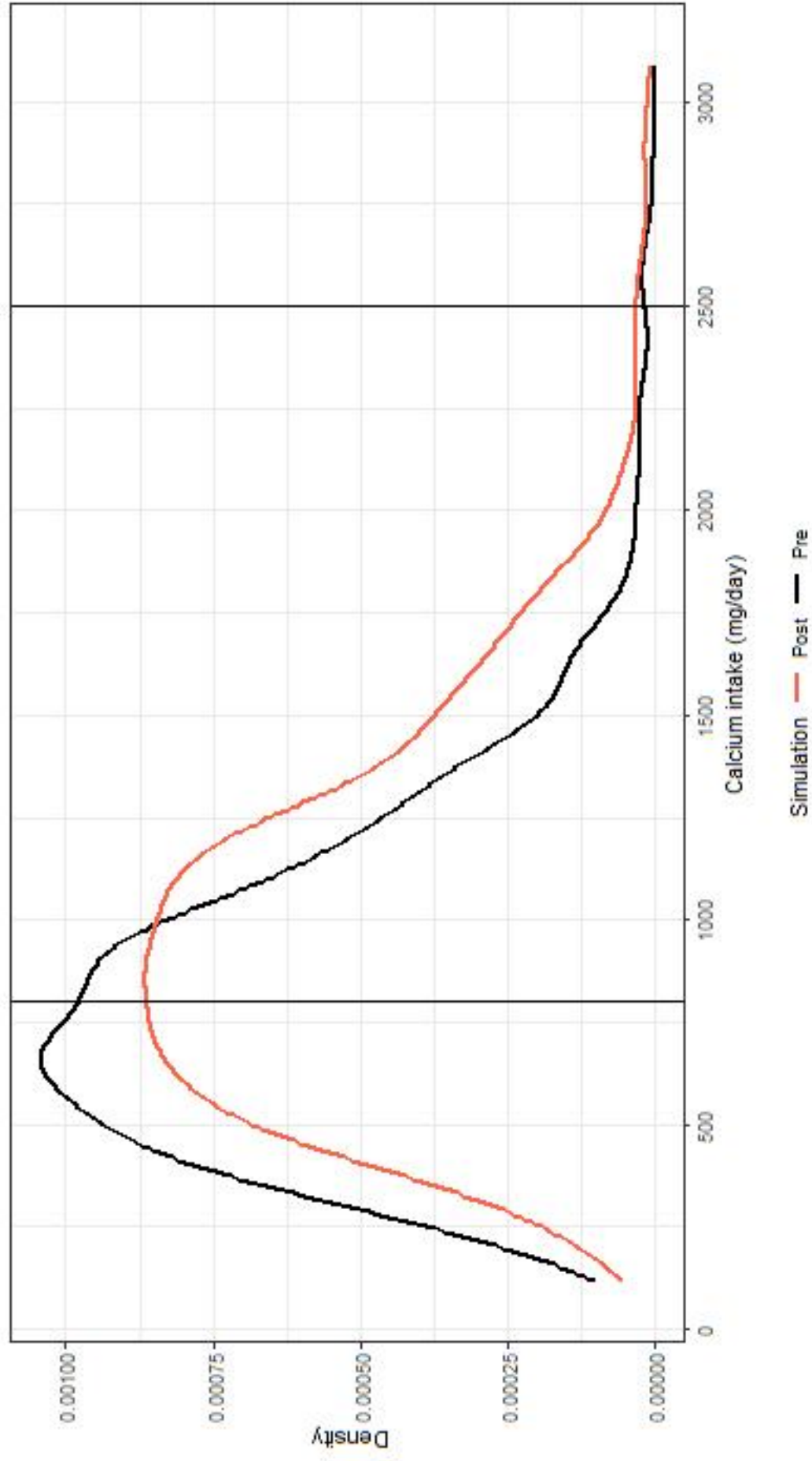

Supplement: Supplementary file 1 — Figure S1. Argentina: distribution of calcium intake pre‐ and post‐flour fortification simulation. Figure S2. Bangladesh: distribution of calcium intake pre‐ and post‐flour fortification simulation. Figure S3. Italy: distribution of calcium intake pre‐ and post‐flour fortification simulation. Figure S4. The Lao PDR: distribution of calcium intake pre‐ and post‐flour fortification simulation. Figure S5. Uganda: distribution of calcium intake pre‐ and post‐flour fortification simulation. Figure S6. The United States: distribution of calcium intake pre‐ and post‐flour fortification simulation. Figure S7. Zambia: distribution of calcium intake pre‐ and post‐flour fortification simulation. [file NYAS-1493-59-s001.zip › Figure S6 - USA Simulation of Flour Fortification.pdf]

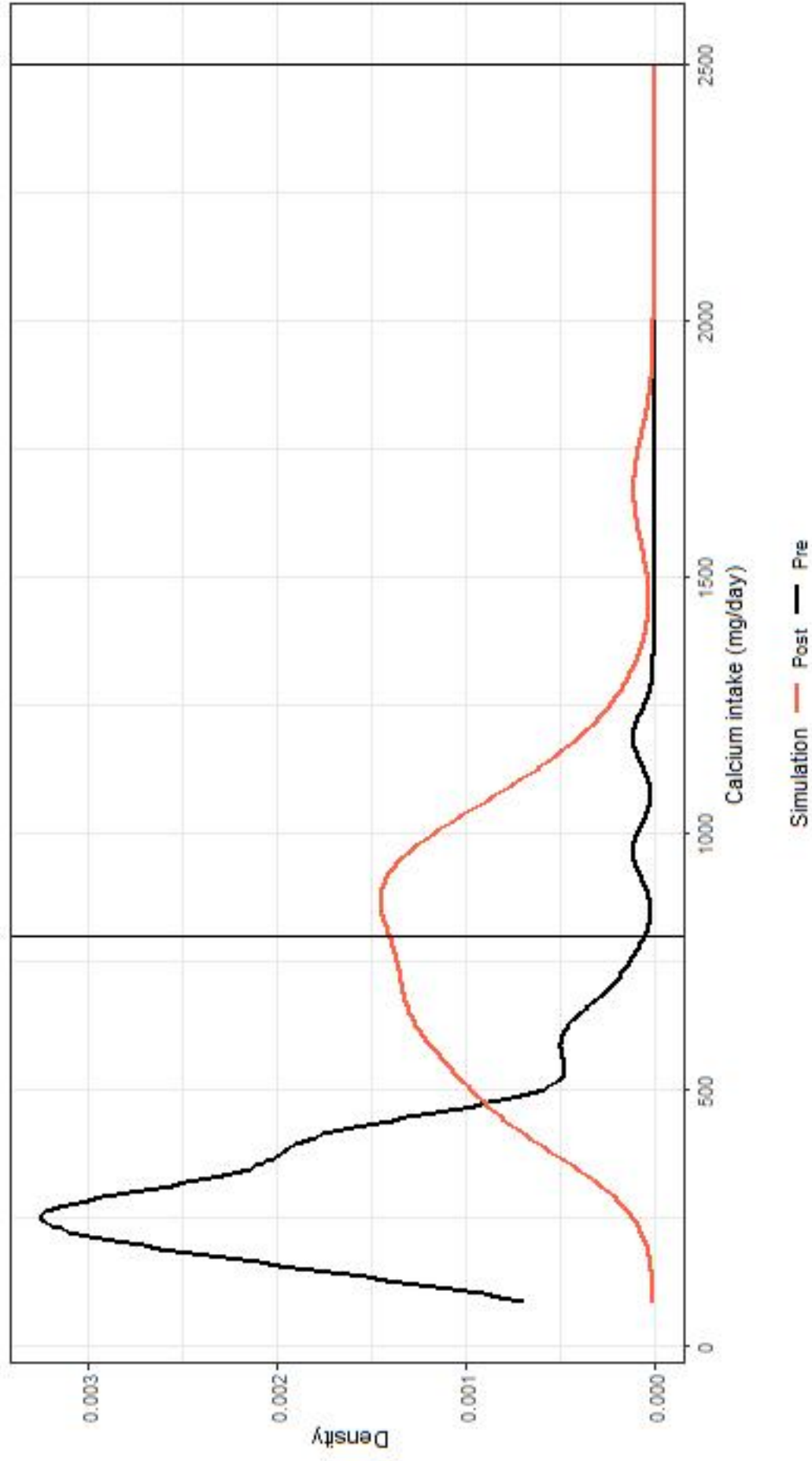

Supplement: Supplementary file 1 — Figure S1. Argentina: distribution of calcium intake pre‐ and post‐flour fortification simulation. Figure S2. Bangladesh: distribution of calcium intake pre‐ and post‐flour fortification simulation. Figure S3. Italy: distribution of calcium intake pre‐ and post‐flour fortification simulation. Figure S4. The Lao PDR: distribution of calcium intake pre‐ and post‐flour fortification simulation. Figure S5. Uganda: distribution of calcium intake pre‐ and post‐flour fortification simulation. Figure S6. The United States: distribution of calcium intake pre‐ and post‐flour fortification simulation. Figure S7. Zambia: distribution of calcium intake pre‐ and post‐flour fortification simulation. [file NYAS-1493-59-s001.zip › Figure S7 - Zambia Simulation of Flour Fortification.pdf]
